# Prevalence of Low Testosterone According to Health Behavior in Older Adults Men

**DOI:** 10.3390/healthcare9010015

**Published:** 2020-12-24

**Authors:** Duk Han Ko, Seong Eon Kim, Ji Young Lee

**Affiliations:** 1 Department of Sports Science Convergence, Dongguk University, Seoul 04620, Korea; kodh119@hanmail.net; 2 Department of Physical Education, Sejong University, Seoul 05006, Korea; 3 Department of Physical Education, Gangneung-Wonju National University, Gangneung 25457, Korea

**Keywords:** testosterone, alcohol drinking, smoking, exercise, healthy lifestyle

## Abstract

Testosterone is a representative sex hormone for men, and low testosterone causes erectile dysfunction and cardiovascular disease. The purpose of this study was to investigate the association between low testosterone (LTT) and health behaviors, such as alcohol, smoking, and exercise habits. We included 2980 men aged 65 to 80. Total serum testosterone and body composition were measured. A testosterone level less than 300 ng/dL was defined as low testosterone. A questionnaire on smoking, alcohol, and exercise was included. The odds ratio (OR) of LTT was calculated through logistic regression. Model 1 only used age as the adjustment variable, whereas Model 2 adjusted for age, waist circumference, and smoking. The prevalence of LTT was 626 (21.0%). The prevalence of LTT was significant in fat mass (Model 1: OR, 2.133) and muscle mass (Model 1: medium OR, 2.130 and low OR, 3.022; Model 2: medium OR, 1.638 and low OR, 1.740). The prevalence of LTT was also different based on smoking (Model 1: OR, 1.590; Model 2: OR, 1.629) and strength exercise (Model 1: OR, 0.849; Model 2: OR, 0.923). In conclusion, high frequency strength exercise and smoking cessation lower the prevalence of low testosterone, and obesity and low muscle mass increase the prevalence of low testosterone.

## 1. Introduction

Testosterone, the sex hormone, is responsible for forming sperm, regulating sexual desire, and expressing secondary sexual characteristics such as muscle and bone growth and body hair [1,2]. Testosterone gradually decreases with age, with a more rapid decrease beginning around age 60 [3,4]. In men, a decrease in testosterone is reported to be associated with sexual problems such as impotence, as well as increased mortality due to insulin resistance, diabetes, hypertension, hyperlipidemia, and cardiovascular disease [5,6,7,8]. Eventually, this decrease in testosterone can reduce quality of life and increase medical burden [9,10]. In addition to the natural decline with aging, multiple factors can affect testosterone levels, including those related to lifestyle habits such as smoking, alcohol, and exercise [11,12,13]. Previous studies report that non-smokers have 4–9% higher testosterone levels than smokers among adults who are at normal weight and do not have diseases such as diabetes, myocardial infarction, heart failure, stroke, or cancer [14]. In a study examining the relationship between alcohol and testosterone, chronic alcohol drinkers had 12% lower testosterone levels than non-drinkers [15]. Finally, a physical activity study found that adult men increased their testosterone levels by 9% through regular aerobic exercise for 12 weeks [16].

Despite many studies on health behavior and testosterone, very few studies have analyzed smoking, alcohol, nutrition, exercise, and body composition simultaneously. Therefore, the purpose of this study was to investigate the relationship between testosterone levels and health behavior characteristics, such as smoking history, exercise habits, and alcohol consumption, using questionnaires. The prevalence of low testosterone (LTT) was calculated by analyzing the influence of health behaviors on LTT. This study established a hypothesis that low alcohol consumption, smoking cessation status, and a high exercise rate would result in a low testosterone prevalence.

## 2. Methods

### 2.1. Participants and Research Progress

**Figure 1:**
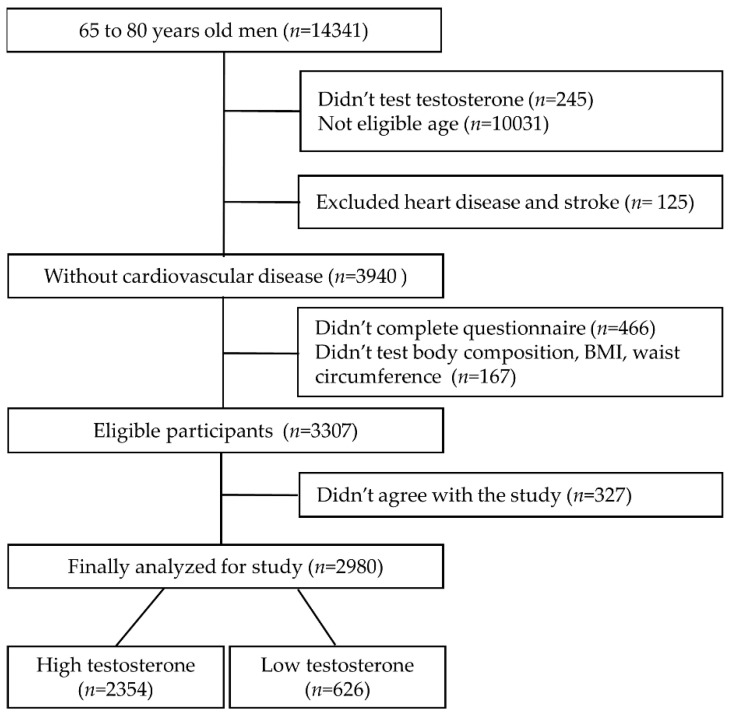
Participant’s inclusion and exclusion criteria diagram.

For the participant data used in this study, a cross-sectional study was conducted using health screening data. A total of 14,341 men aged 65–80 years tested from 2012 to 2017 at a hospital in South Korea were included. The inclusion criteria were an agreement to participate in the study and completion of all tests for the purpose of the study. Exclusion criteria were as follows: not tested for testosterone (*n* = 245), under 65 or over 80 years of age (*n* = 10,031), and diagnosis of ischemic heart disease or stroke (*n* = 125). Additionally, 466 men who did not complete the questionnaire and 167 who did not undergo waist circumference or body mass index (BMI) and body composition measurement were excluded. Finally, 327 people who disagreed with the use of their results for the study were excluded. Therefore, 2980 people were included in the analysis (Figure 1).

Participants were asked to visit the hospital before 8 a.m., where they showed identification and underwent the medical examination. Participants filled out a medical history and health behavior questionnaire and were provided with gowns and slippers from the testing agency. Patients first had their height, weight, and waist circumference measured, and then blood-collecting and urine tests were performed to exclude the effect of physical activity on these parameters.

Before blood tests, individuals fasted for 8 h. The total serum testosterone level was measured through blood collection through the median cubital vein. In this study, testosterone ≤ 300 ng/dL was defined as LTT and testosterone > 300 ng/dL as high testosterone (HTT), as in previous studies [17]. All participants agreed to participate in the study, and this study was approved by the Institutional Review Board (c).

### 2.2. Health Behavior Questionnaire (Alcohol, Smoking, and Exercise)

The participants were surveyed about smoking, alcohol consumption, and exercise habits through an internet questionnaire before the medical test. The health behavior questionnaire was constructed based on the World Health Organization (WHO) Alcohol, Smoking and Substance Involvement Screening Test [18]. Current smokers were defined as the “current” group, those who quit smoking as the “former” group, and those who had never smoked at all as the “never” group. To assess alcohol consumption, the pure alcohol amount was calculated according to the WHO guidelines by investigating the type, frequency, and amount of alcohol consumption. Here, 1–40 g/day was defined as “low risk,” 41–60 g/day as “medium risk,” and >61 g/day as “high risk” [19]. To assess exercise, we used the WHO’s International Physical Activity Questionnaire to investigate specific types of exercise. Additional questionnaires were conducted on the type of exercise (aerobic, strength exercise, or stretching), frequency of exercise, and duration of one session [20]. The American College of Sports Medicine (ACSM) guidelines were used to define exercise frequency [21]. For healthy adults, the ACSM recommends aerobic exercise at least 3 days a week and strength exercise and stretching at least 2 days a week. Therefore, in this study, 0–2 days of aerobic exercise a week was defined as “low,” 3–4 days as “medium,” and 5–7 days as “high.” For strength exercise and stretching, 0–1 days a week was defined as “low,” 2–4 days as “medium,” and 5–7 days as “high”.

### 2.3. Body Composition and Anthropometric Measurement

Body measurement was performed using the Inbody 720 (Biospace Co., Seoul, Korea), and body composition was analyzed using the impedance method. In this study, percent body fat and percent skeletal muscle were used as variables. Percent body fat within 22% is recommended for optimal health by the ACSM [21]. Therefore, body fat ≤ 22.0% was classified as normal and body fat ≥ 22.1% was classified as obese. Skeletal muscle mass was classified as low, medium, or high by tertile. BMI and waist circumference were classified according to the WHO’s Asia-Pacific criteria [22]. A BMI ≤ 22.9 kg/m^2^ was classified as “normal,” a BMI of 23.0–24.9 kg/m^2^ as “overweight,” and a BMI ≥ 25.0 kg/m^2^ as “obese.” A waist circumference ≤ 89.9 cm was defined as “normal,” and a waist circumference ≥ 90.0 cm as “obese.”

### 2.4. Data Analysis

Data analysis was performed using the statistical program SPSS 25.0 (SPSS IBM Corp., Armonk, NY, USA). Continuous variables such as height, weight, BMI, body fat percentage, and muscle mass are expressed as mean and standard deviation (mean ± SD), and an independent *t*-test was performed to compare the HTT and LTT groups. In order to perform logistic regression analysis, continuous variables were converted into categories, and Chi-square test was performed to assess differences in categorical variables. Logistic regression analysis was used to evaluate the impact of different health behavior factors on LTT. Results are presented as odds ratio (OR) and 95% confidence intervals. The reference value was set to the “low risk” alcohol and “never” smoking groups, and the “low” frequencies were used as references for exercise-related factors. As testosterone is affected by age, Model 1 used only age as the adjustment variable. For Model 2, multiple logistic regression analysis and stepwise selection were performed to identify significant variables in addition to age, and waist circumference and smoking were added. The statistical significance level was set to *p* < 0.05.

## 3. Results

### 3.1. Participant Characteristics

Table 1 and Table 2 show the general characteristics of the participants. HTT and LTT patients were 2354 and 626, respectively. Significant differences between the two groups were found in weight, BMI, waist circumference, fat mass, and muscle mass, but no significant differences in age (*p* = 0.127) and height (*p* = 0.340) (Table 1).

Table 2 shows the comparison of categorized variables between groups. BMI, waist circumference, fat mass, muscle mass, smoking, and strength exercise were significant between groups. However, non-significant results were found between groups in alcohol consumption (*p* = 0.699) and stretching (*p* = 0.560) (Table 2).

### 3.2. Low Testosterone Prevalence According to BMI, Waist Circumference and Body Composition

Table 3 shows the LTT prevalence based on body composition and anthropometric variables. The OR for BMI was 1.612 in the obese group (compared to the normal BMI group) in Model 2, but the OR for BMI was not significant in the overweight group. For waist circumference, in Model 2, the OR was 2.060 in the obese group (compared to the normal BMI group). Body fat mass had a significant OR in Model 1 but not in Model 2. Muscle mass was classified into tertile groups. The ORs for LTT were 1.638 and 1.740 in the medium and low groups, respectively, compared to the high group.

### 3.3. Low Testosterone Prevalence According to Alcohol Risk, Smoking Status and Exercise Habit

**Figure 2:**
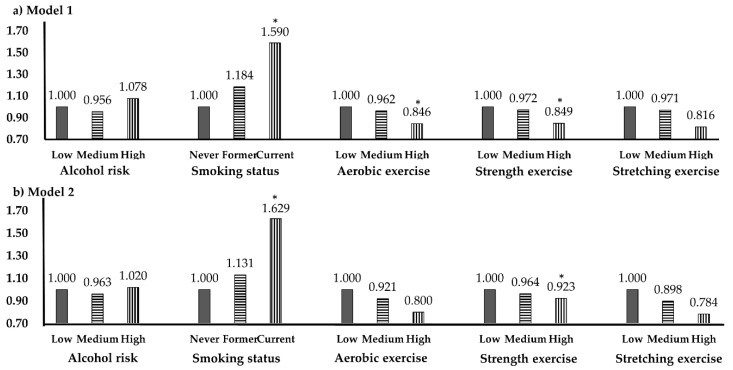
Low testosterone prevalence according to health behavior. * *p* < 0.05; OR, odds ratio; CI, confidence interval. (**a**) Model 1, adjusted variable is age. (**b**) Model 2, adjusted variables are age, waist circumference, smoking.

Figure 2 shows the LTT prevalence according to health behavior. In Model 1, which was adjusted only for age, significant OR values appeared in smoking, aerobic exercise, and strength exercise, but in Model 2, significant OR values were derived only for smoking and strength exercise. For smoking, the age-adjusted ORs for current smokers were 1.590 (*p* = 0.012) in Model 1 and 1.629 (*p* = 0.010) in Model 2. For aerobic exercise, the age-adjusted OR in the highest frequency group was 0.846 (*p* = 0.043). The ORs for strength exercise were 0.849 (*p* = 0.042) in Model 1 and 0.923 (*p* = 0.022) in Model 2.

## 4. Discussion

Decreased testosterone is often reported in people at risk for obesity and cardiovascular disease and is common with aging [20,23]. One study reported LTT in more than 20% of individuals in their 60s and more than 30% of individuals in their 70s [24,25]. Exercise is known to have a positive effect on testosterone secretion [16]. This study was a cross-sectional analysis to investigate and health behavior, obesity, body composition and LTT prevalence in a relatively large number of older men. We found that BMI, waist circumference, body fat percentage, muscle mass, smoking, and exercise, especially strength exercise, had an influence on LTT.

Akishita’s study reported that LTT was associated with metabolic risk factors such as blood pressure, triglycerides, and blood sugar levels, and abdominal obesity had the highest association with these factors [26]. Similar to the previous study, the LTT group had a significantly higher BMI, waist circumference, and fat percentage than the HTT group in this study. Also, a study examining the association of obesity and testosterone level in middle-aged 40–60 years old showed similar results. The group with a waist circumference ≥ 100 cm had statistically significantly lower testosterone levels than the group with a waist circumference < 100 cm, and individuals with a BMI of 25 kg/m^2^ or more had a statistically significantly lower testosterone level than those with a BMI less than 25 kg/m^2^ [27]. Among individuals with obesity, visceral fat activates adipose tissue to make large amounts of adipokines and cytokines, such as tumor necrosis factor α, interleukin (IL)-6, and IL-8. These factors play an important role in maintaining low levels of inflammation. However, they can also cause endocrine dysfunction, such as increasing insulin resistance and decreasing metabolic function. This result is associated with low levels of sex hormone binding globulin, which regulates testosterone levels [28].

In this study, current smokers were found to have a high prevalence of LTT. A previous study compared testosterone levels between current smokers, past smokers, and non-smokers in 426 individuals. Current smokers had lower testosterone levels than non-smokers by an average of −0.61 ± 0.23 ng/dL [29]. Therefore, even if you are a smoker, smoking cessation has the potential to prevent LTT. Another finding of this study, prevalence of LTT was not affected by risk of alcohol consumption. These results were similar to a study of smoking, alcohol consumption and testosterone in 1563 men. The results indicated that smoking was an independent factor affecting testosterone levels; however, alcohol consumption was not significantly associated with testosterone [30].

The prevalence of LTT showed significant values in aerobic and strength exercise, but not in stretching. Hayes et al. [31] reported that older men (62 ± 2 years) in the United Kingdom who underwent high-intensity exercise for 6 weeks had a 17% increase in testosterone levels compared to men who did not exercise [31]. In another study related to high strength, an experiment was performed using a bicycle. They increased the load at 15 watts per minute while maintaining 60 RPM for as long as the participant was able. In the results, testosterone increased by 34% with high-intensity exercise experiment using a bicycle [32]. These results suggest that high-intensity exercise has a positive effect on the increase in testosterone. However, some studies have reported that low-intensity exercise is also positive for testosterone. Khoo’s study showed a statistically significant increase (17%) in the total testosterone levels as a result of a 24-week low-intensity aerobic exercise regimen in obese men under 44 years of age [33]. Aerobic exercise and strength exercise have shown positive results for testosterone in other studies [34], but further research is needed to determine which exercise is more effective.

A unique feature of this study was that exercise was classified into aerobic exercise, strength exercise, and stretching, and each type of exercise was further classified according to exercise frequency per week. In the results, participants who had high rate aerobic and strength exercised had significantly lower LTT prevalence. Meanwhile, stretching exercise was not associated with LTT prevalence. Strength exercise causes many changes and adaptation mechanisms in the neuroendocrine system, such as the release of anabolic hormones, including growth hormone, insulin-like growth factor 1, and androgen, and catabolic hormones, including cortisol [35]. Testosterone, which is a type of androgen, interacts with skeletal muscle to stimulate protein synthesis and regulate the ratio of muscle to fat [36]. It is likely that strength exercise influences testosterone levels by this mechanism. However, this study did not consider the intensity of exercise, and because this study used a questionnaire filled out by the individual, a simple comparison with previous studies may not be possible. This study may provide a useful background in providing realistic advice on lifestyle improvements to prevent or resolve LTT in the elderly. In particular, it can emphasize the necessity of strength exercise to not only relieve obesity but also increase muscle mass, and explain the benefits of smoking cessation to smokers.

This study has several limitations. First, since it was targeted to patients undergoing medical examination, the testosterone levels were expected to change if the individual was administered medication for diabetes, blood pressure, hyperlipidemia, or cardiovascular disease at the time of the investigation. However, this classification was not possible. Second, we must also consider the validity of the questionnaire. In particular, it should be considered that certain objective criteria may be insufficient because the patient’s self-administered questionnaire relied on the past and present disease state as exclusion criteria. Last, because it was a cross-sectional study, the universality of the results could not be confirmed. Therefore, intervention and longitudinal studies on testosterone level changes with long-term follow-up are needed.

This study has limitations in explaining that obesity and health behavior affect testosterone levels, because this study was carried out as a cross-sectional design. For example, because low testosterone leads to low muscle mass, it cannot be ruled out that they avoid strength exercise. In the future, the study of longitudinal design will be able to contribute more to solving this problem. In addition, testosterone levels were lower in subjects who were obese in BMI and waist circumference. This is also related to exercise, but diet considerations have not been made. Therefore, further research is needed to investigate the relationship between dietary intake, race, occupation, income, marital status, and stress and testosterone.

## 5. Conclusions

In conclusion, body factors such as BMI, waist circumference, muscle mass, and body fat percentage were significantly associated with low testosterone among men aged 65 to 80. Lifestyle factors such as aerobic exercise and strength exercise also had an effect on low testosterone prevention. However, smoking and obesity increased low testosterone. For aerobic and strength exercise, 5 to 7 days per week had the greatest effect on testosterone. Therefore, for the management of testosterone levels, it is important to maintain a normal body weight by smoking cessation and frequent strength exercise.

## Figures and Tables

**Table 1:** Comparison between high testosterone and low testosterone.

Variables	HTT (*n* = 2354)	LTT (*n* = 626)	*p*
Age, years	68.8 ± 4.9	69.2 ± 4.6	0.127
Height, cm	171.2 ± 5.6	171.5 ± 5.7	0.340
Weight, kg	72.5 ± 9.1	76.5 ± 9.5	<0.001 *
Body mass index, kg/m^2^	24.7 ± 2.6	26.0 ± 2.6	<0.001 *
Waist circumference, cm	88.0 ± 7.2	91.8 ± 7.2	<0.001 *
Fat mass, %	22.3 ± 4.8	24.7 ± 4.6	<0.001 *
Muscle mass, %	43.7 ± 2.9	42.3 ± 2.8	<0.001 *
Testosterone, ng/dL	544 ± 145	244 ± 125	<0.001 *

* *p* < 0.05; values expressed as mean ± SD; it was analyzed with independent *t*-test. HTT, high testosterone; LTT, low testosterone.

**Table 2:** High testosterone and low testosterone ratio of categorized variables.

Variables	Groups	HTT (*n* = 2354)	LTT (*n* = 626)	*p*
Body mass index	Normal	526 (22.3%)	56 (8.9%)	<0.001 *
	Overweight	804 (34.2%)	196 (31.3%)	
	Obese	1024 (43.5%)	374 (59.8%)	
Waist circumference	Normal	1420 (60.3%)	270 (43.1%)	<0.001 *
	Obese	934 (39.7%)	356 (56.9%)	
Fat mass	Low	1134 (48.2%)	190 (30.4%)	<0.001 *
	High	1220 (51.8%)	436 (69.6%)	
Muscle mass	High	912 (38.7%)	124 (19.8%)	<0.001 *
	Medium	746 (31.7%)	216 (34.5%)	
	Low	696 (29.6%)	286 (45.7%)	
Alcohol consumption	Low risk	454 (19.3%)	120 (19.2%)	0.699
	Medium risk	1100 (46.7%)	278 (44.4%)	
	High risk	800 (34.0%)	228 (36.4%)	
Smoking status	Never	1368 (58.1%)	308 (49.3%)	0.016 *
	Former	647 (27.5%)	196 (31.3%)	
	Current	339 (14.4%)	122 (19.4%)	
Aerobic exercise	Low	1160 (49.3%)	336 (53.7%)	0.036 *
	Medium	862 (36.6%)	233 (37.2%)	
	High	332 (14.1%)	57 (9.1%)	
Strength exercise	Low	1071 (45.5%)	306 (48.8%)	0.034 *
	Medium	1062 (45.1%)	286 (45.7%)	
	High	221 (9.4%)	34 (5.5%)	
Stretching exercise	Low	646 (27.4%)	188 (29.9%)	0.560
	Medium	1300 (55.2%)	316 (50.6%)	
	High	408 (17.4%)	122 (19.5%)	

* *p* < 0.05; values expressed as number (%); it was analyzed with Chi-square test; HTT, high testosterone; LTT, low testosterone.

**Table 3:** Low testosterone prevalence according to BMI, waist circumference and body composition.

		Model 1		Model 2	
Variables	Classification	OR (95% CI)	*p*	OR (95% CI)	*p*
Body mass index	Normal	Reference	-	Reference	-
	Overweight	2.290 (1.463–3.583)	<0.001 *	1.426 (0.825–2.466)	0.204
	Obese	3.431 (2.245–5.243)	<0.001 *	1.612 (1.005–2.584)	0.048 *
Waist circumference	Normal	Reference	-	Reference	-
	Obese	2.005 (1.558–2.580)	<0.001 *	2.060 (1.596–2.658)	<0.001 *
Fat mass, %	Low	Reference	-	Reference	-
	High	2.133 (1.634–2.785)	<0.001 *	1.299 (0.945–1.786)	0.107
Muscle mass, %	High	Reference	-	Reference	-
	Medium	2.130 (1.514–2.995)	<0.001 *	1.638 (1.142–2.350)	0.007 *
	Low	3.022 (2.175–4.200)	<0.001 *	1.740 (1.174–2.578)	0.006 *

* *p* < 0.05; OR, odds ratio; CI, confidence interval. Model 1, adjusted variable is age. Model 2, adjusted variables are age, waist circumference, smoking.

## Data Availability

The data are not publicly available due to privacy or ethical.
